# Social Deprivation as a Driver of Disparities in Arthroplasty: A Contemporary Review

**DOI:** 10.1007/s12178-026-10046-7

**Published:** 2026-06-19

**Authors:** Rimel N. Mwamba, Shannon Stover, Anna Lavernia, Rachel Bergman, Linda Suleiman

**Affiliations:** 1 The Pritzker School of Medicine, The University of Chicago Medical Center, Chicago, IL 60637 USA; 2 University of Notre Dame, Holy Cross Dr, Notre Dame, IN 46556 USA; 3 The University of Chicago, 5801 S Ellis Ave, Chicago, IL 60637 USA; 4 Department of Orthopaedic Surgery, Northwestern University, 259 E. Erie St. 13th Floor, Chicago, IL 60611 USA

**Keywords:** Social deprivation, Neighborhood disadvantage, Socioeconomic status, Total knee arthroplasty, Total hip arthroplasty, Arthroplasty outcomes

## Abstract

**Purpose of Review:**

While social determinants of health are known to contribute to disparities in orthopaedic care, associations between social deprivation and total joint arthroplasty (TJA) outcomes remain unclear. This review assessed the relationship between social deprivation and multiple aspects of total joint arthroplasty, including access to care, early postoperative outcomes, resource utilization, and patient-reported functional recovery.

**Recent Findings:**

Recent studies have increasingly used composite deprivation indices including the Area Deprivation Index (ADI), Social Vulnerability Index (SVI), Social Deprivation Index (SDI), and Distressed Communities Index (DCI) to examine disparities in TJA care. Higher levels of social deprivation were consistently associated with reduced arthroplasty utilization, increased early postoperative complications, longer hospital stays, higher costs, and greater likelihood of non-home discharge. Associations with emergency department use were frequent, whereas findings related to readmissions were mixed. Relationships between deprivation and patient-reported outcomes were less consistent. Across multiple studies, deprivation was not uniformly associated with failure to achieve clinically meaningful improvement but was more frequently linked to failure to achieve patient-acceptable symptom states and challenges in sustaining functional recovery.

**Summary:**

Social deprivation is an important determinant of access, perioperative outcomes, and healthcare utilization following TJA, with ADI demonstrating the most consistent association. Although socially deprived patients generally achieve meaningful postoperative improvement, they are less likely to reach optimal recovery thresholds. Incorporating deprivation measures into perioperative planning may help identify patients who would benefit from targeted interventions. Future studies should standardize index use and evaluate strategies to reduce socioeconomic disparities in arthroplasty outcomes.

## Introduction

Total joint arthroplasty (TJA) has experienced substantial growth in utilization worldwide and is an effective intervention for improving pain, function, and quality of life in patients with advanced degenerative joint disease [1]. Despite formidable success, significant disparities persist in pre- and postoperative outcomes across patient populations [2]. Recent literature has explored how social deprivation indices, measures of socioeconomic disadvantage, highlight disparate access to TJA and outcomes following surgical intervention.

Social deprivation indices are methodological tools designed to elucidate socioeconomic status using measures of income, education, employment, and housing quality [3]. The area deprivation index (ADI), social vulnerability index (SVI), social deprivation index (SDI), and distressed community index (DCI) have received increased attention in recent years to characterize socioeconomic disadvantage with a greater detail than traditional single-variable measures such as income, insurance status, or educational attainment [4–7]. Despite increasing interest, studies examining the relationship between social deprivation and TJA have reported variable findings. These findings may better reflect true outcome differences or variability introduced by the use of different social deprivation indices. Given these inconsistencies, a review of the existing literature may help clarify how social deprivation relates to utilization, complications, and readmissions after TJA and provide greater clarity for current practice guidelines.

This study aims to evaluate the association between social deprivation and pre- and postoperative care and outcomes in patients undergoing TJA. Specifically, we aim to examine associations between social deprivation and access to TJA, early postoperative clinical outcomes, healthcare utilization, and patient-reported outcomes of functional recovery. Ultimately, this work seeks to inform the integration of social risk into arthroplasty care pathways and support efforts to reduce socioeconomic disparities in TJA outcomes.

## Methods

### Search Strategy

A comprehensive review of PubMed, Cochrane Library, and EMBASE was performed to identify studies that assessed various indices of social deprivation in total knee arthroplasty (TKA), and total hip arthroplasty (THA). The search utilized a combination of keywords and MeSH terms including: joint replacement, joint arthroplasty, THA, TKA, TJA, social deprivation, area deprivation, social vulnerability, and distressed communities. To assess outcomes, additional terms included were: complication, infection, re-admission, revision, length of stay, discharge and patient-reported-outcome-measures. The search was conducted on November 1, 2025 and included articles between January 1, 2020 and October 31, 2025.

### Eligibility and Study Selection

Studies were eligible for inclusion if they were English-language, full-text articles evaluating TKA and/or THA in the United States and examined associations between social deprivation and surgical access, perioperative outcomes, or patient-reported outcome measures. Case reports, case series, conference abstracts, and animal studies were excluded.

Study selection followed the Preferred Reporting Items for Systematic Reviews and Meta-Analyses (PRISMA) framework for this contemporary review. Two authors (SS and AL) independently screened titles and abstracts and assessed full-text eligibility. Discrepancies were resolved through review by a third independent reviewer (RM). Following removal of duplicates and initial screening, 42 studies underwent full-text review, of which 34 met inclusion criteria and were included in final analysis (Table 1).

**Table 1:** All studies included in the final analysis (*n* = 34)

Study ID	First author		Year	Procedure	Deprivation Index
1	Jeremy A Dubin	The Utility of the Social Vulnerability Index as a Proxy for Social Disparities Following Total Knee Arthroplasty	2024	Total knee arthroplasty	Social vulnerability index (SVI)
2	Adam M Gordon	A Higher Area Deprivation Index Is Associated with Increased Medical Complications and Emergency Department Utilizations After Total Hip Arthroplasty	2025	Total hip arthroplasty	Area deprivation index (ADI)
3	Adam M Gordon	A Nationwide Analysis of the Impact of Socioeconomic Status on Complications and Health Care Utilizations After Total Knee Arthroplasty Using the Area Deprivation Index: Consideration of the Disadvantaged Patient	2024	Total knee arthroplasty	Area deprivation index (ADI)
4	Muyibat A Adelani	Are Neighborhood Characteristics Associated With Outcomes After THA and TKA? Findings From a Large Healthcare System Database	2023	Total knee arthroplasty; Total hip arthroplasty	Area deprivation index (ADI)
5	Michelle R Shimizu	Are social determinants of health associated with an increased length of hospitalization after revision total hip and knee arthroplasty? A comparison study of social deprivation indices	2024	Revision total knee arthroplasty; Revision total hip arthroplasty	Area deprivation index (ADI); Social deprivation index (SDI), and Social vulnerability index (SVI)
6	Jeremy Dubin	Assessing social disparities in inpatient vs. outpatient arthroplasty: a in-state database analysis	2024	All total joint arthroplasty	Social vulnerability index (SVI)
7	Sarah H Yi	Community-Level Social Vulnerability and Hip and Knee Joint Replacement Surgery Receipt Among Medicare Enrollees With Arthritis	2024	Total knee arthroplasty; Total hip arthroplasty	Minority health social vulnerability index (MH-SVI)
8	Kimberly A Rollings	Comparing Deprivation vs. Vulnerability Index Performance Using Medicare Beneficiary Surgical Outcomes	2024	Total knee arthroplasty; Total hip arthroplasty	Area deprivation index (ADI); Social vulnerability index (SVI)
9	Jonathan H Shaw	Comparison of Area Deprivation Index, Socioeconomic Parameters, and Preoperative Demographics With Postoperative Emergency Department Visits After Total Knee Arthroplasty	2021	Total knee arthroplalsty	Area deprivation index (ADI)
10	Michelle R Shimizu	The Utility of Neighborhood Social Vulnerability Indices in Predicting Non-Home Discharge Disposition Following Revision Total Joint Arthroplasty: A Comparison Study	2025	Revision total knee arthroplasty; Revision total hip arthroplasty	Area deprivation index (ADI); Social deprivation index (SDI); Social vulnerability index (SVI)
11	Tahsin M Rhman	The Impact of Social Determinants of Health on Outcomes and Complications After Total Knee Arthroplasty: An Analysis of Neighborhood Deprivation Indices	2024	Total knee arthroplasty	Area deprivation index (ADI); Social vulnerability index (SVI)
12	Lulla V Kiwinda	The Effect of Social Drivers of Health on 90-Day Readmission Rates and Costs After Primary Total Hip and Total Knee Arthroplasty	2025	Total knee arthroplasty; Total hip arthroplasty	Area deprivation index (ADI)
13	Anoop S Chandrashekar	Socioeconomic Indices Are Associated With Increased Resource Utilizations, but Not 90-Day Complications Following Total Hip and Knee Arthroplasty	2025	Total knee arthroplasty; Total hip arthroplasty	Area deprivation index (ADI); Distressed community index (DCI)
14	Nicholas R Pagani	Socioeconomic Disadvantage Predicts Decreased Likelihood of Maintaining a Functional Knee Arthroplasty Following Treatment for Prosthetic Joint Infection	2024	Revision total knee arthroplasty	Area deprivation index (ADI)
15	Samantha N Baxter	Social Vulnerability adversely affects emergency-department utilization but not patient-reported outcomes after total joint arthroplasty	2024	Total knee arthroplasty; Total hip arthroplasty	Social vulnerability index (SVI)
16	Elizabeth C Danielson	Social risk and patient-reported outcomes after total knee replacement: Implications for Medicare policy	2024	Total knee arthroplasty	Social vulnerability index (SVI)
17	Allen C Norris	Social Needs of Patients Undergoing Total Joint Arthroplasty	2022	Total knee arthroplasty; Total hip arthroplasty	Area deprivation index (ADI)
18	Katherine Hadlandsmyth	Social Determinants of Long-Term Opioid Use Following Total Knee Arthroplasty	2024	Total knee arthroplasty	Area deprivation index (ADI)
19	Ronald E Delanois	Social Determinants of Health in Total Hip Arthroplasty: Are They Associated With Costs, Lengths of Stay, and Patient Reported Outcomes?	2022	Total hip arthroplasty	Social vulnerability index (SVI)
20	Joydeep Baidya	Social determinants of health in patients undergoing hemiarthroplasty: are they associated with medical complications, healthcare utilization, and payments for care?	2023	Hemiarthroplasty	Area deprivation index (ADI)
21	Matthew J Hadad	Racial Disparities in Outcomes After THA and TKA Are Substantially Mediated by Socioeconomic Disadvantage Both in Black and White Patients	2023	Total knee arthroplasty; Total hip arthroplasty	Area deprivation index (ADI)
22	Jeremy A Dubin	Race Associated With Increased Complication Rates After Total Knee Arthroplasty	2023	Total knee arthroplasty	Area deprivation index (ADI)
24	Anton Khlopas	Neighborhood Socioeconomic Disadvantages Associated With Prolonged Lengths of Stay, Nonhome Discharges, and 90-Day Readmissions After Total Knee Arthroplasty	2022	Total knee arthroplasty	Area deprivation index (ADI)
25	Sandeep S Bains	Neighborhood socioeconomic disadvantages associated with increased rates of revisions, readmissions, and complications after total joint arthroplasty	2024	Total knee arthroplasty; total hip arthroplasty	Area deprivation index (ADI)
26	Brian Benyamini	Neighborhood Socioeconomic Disadvantage May Influence 1-Year Patient-Reported Outcome Measures After Total Hip Arthroplasty	2025	Total hip arthroplasty	Area deprivation index (ADI)
27	Benjamin E Jevnikar	Neighborhood Socioeconomic Disadvantage is Associated With Increased Health Care Utilization After Septic and Aseptic Revision Total Hip Arthroplasty	2025	Revision total hip arthroplasty	Area deprivation index (ADI)
28	Daniel Grits	Neighborhood Socioeconomic Disadvantage Associated With Increased Healthcare Utilization After Total Hip Arthroplasty	2022	Total hip arthroplasty	Area deprivation index (ADI)
29	Juan D Lizcano	Health Disparities in Aseptic Revision Total Hip Arthroplasty: Assessing the Impact of Social Determinants of Health	2025	Revision total hip arthroplasty	Area deprivation index (ADI); Social vulnerability index (SVI)
30	Matthew J Hadad	High Area Deprivation Index is Associated With Not Achieving the Patient-acceptable Symptom State After TKA	2024	Total knee arthroplasty	Area deprivation index (ADI)
31	Genna R Potter	How Many Patients Qualify for Extended Oral Antibiotic Prophylaxis Infection Following Primary and Revision Hip and Knee Arthroplasties	2024	Revision total knee arthroplasty; Revision total hip arthroplasty	Area deprivation index (ADI)
32	Pedro J Rullan	How to Raise the Bar in the Capture of Patient-Reported Outcome Measures in Total Joint Arthroplasty	2024	Total knee arthroplasty; Total hip arthroplasty	Area deprivation index (ADI)
33	Victoria S Wu	Impact of social disadvantage among total knee arthroplasty places of service on procedural volume: a nationwide Medicare analysis	2023	Total knee arthroplasty	Area deprivation index (ADI)
34	Davis A Hartnett	Socioeconomic Disparities in the Utilization of Total Hip Arthroplasty	2022	Total hip arthroplasty	Social deprivation index (SDI)

### Data Extraction

For each included study, data were extracted on publication characteristics, including title, authorship, and year of publication. Information regarding the social deprivation index used such as ADI, SVI, SDI and DCI was recorded. Details on arthroplasty type, including total knee arthroplasty and/or total hip arthroplasty, were obtained. Outcomes of interest included measures of access, perioperative outcomes, and patient-reported outcomes.

## Results

### Social Deprivation Measurements in Arthroplasty Research

This study assessed the association of social deprivation and total joint arthroplasty. Our review demonstrated that four deprivation indices were used in the literature: ADI, SVI, SDI, and DCI. ADI is a neighborhood-level measure of socioeconomic disadvantage derived from U.S. Census data. This metric incorporates 17 variables across income, education, employment and housing quality. SVI is a composite, area-based measure developed by the U.S. Centers for Disease Control and Prevention (CDC) to assess a community’s vulnerability to external stressors. This metric incorporates 15 variables across socioeconomic status, household composition and disability, minority status and language, as well as housing type and transportation across census tract levels. SDI serves as a composite measure of area-level socioeconomic deprivation that uses 7 variables to quantify social conditions associated with poor health outcomes, typically at the ZIP code or census tract level. DCI is an economic well-being measure that evaluates local economic distress using seven indicators that classify communities from prosperous to distressed.

Of the 34 articles assessed in the review, 26 articles (76%) used area deprivation index as a measure for social deprivation; 10 articles (29%) used social vulnerability index as a measure of social deprivation; 2 articles (5%) used social deprivation index as a measure of social deprivation, and 1 article (3%) used distressed community index as a measure of social deprivation (Table 1).

### Access to Arthroplasty and 30–90 Day Clinical Outcomes

In this review, 20 studies were identified which assessed access to arthroplasty and early postoperative outcomes. The majority of these studies demonstrated that high levels of social deprivation were associated with reduced arthroplasty utilization and worse 30–90-day outcomes, though findings were heterogeneous across indices (Table 2). Two articles reported that receipt of arthroplasty and procedural volume was much lower in more deprived communities [8, 9]. In Medicare analyses, total knee arthroplasty utilization also decreased with increasing deprivation at the place of service level [8]. Reduced odds of total hip arthroplasty utilization were observed among patients with greater socioeconomic disadvantage despite hip osteoarthritis diagnoses [9].

**Table 2:** Studies assessing social deprivation and access to arthroplasty and 30–90 day clinical outcomes in patients undergoing total joint arthroplasty (*n* = 20)

First author		Year	Key Findings
Jeremy A Dubin	The Utility of the Social Vulnerability Index as a Proxy for Social Disparities Following Total Knee Arthroplasty	2024	■ Household composition and disability was a risk factor for total complications ■ Patients with an SVI > 0.5 were more likely to be women, Black and have a median household income of <$87,999
Adam M Gordon	A Higher Area Deprivation Index Is Associated with Increased Medical Complications and Emergency Department Utilizations After Total Hip Arthroplasty	2025	■ Patients undergoing THA who had high ADI had higher rate and odds of developing any medical complications including acute kidney injuries, myocardial infarctions and surgical site infections ■ Patients with high ADI had higher rates and odds of ED visits within 90 days but no significant difference in readmissions
Adam M Gordon	A Nationwide Analysis of the Impact of Socioeconomic Status on Complications and Health Care Utilizations After Total Knee Arthroplasty Using the Area Deprivation Index: Consideration of the Disadvantaged Patient	2024	■ Patients with high ADI had higher rates and odds of any medical complications, respiratory failures, and acute kidney injuries ■ Patients with higher ADI had lower readmission rates but higher 90-day ED visits
Kimberly A Rollings	Comparing Deprivation vs. Vulnerability Index Performance Using Medicare Beneficiary Surgical Outcomes	2024	■ Patients with higher levels of ADI and SVI had significantly greater odds of unplanned surgery, 30-day readmissions and 30-day mortality ■ Patients with higher SVI, compared to ADI, had larger outcome rate differences
Jonathan H Shaw	Comparison of Area Deprivation Index, Socioeconomic Parameters, and Preoperative Demographics With Postoperative Emergency Department Visits After Total Knee Arthroplasty	2021	■ There was no difference in ED admissions within 90 days for patients with low and higher ADI
Tahsin M Rhman	The Impact of Social Determinants of Health on Outcomes and Complications After Total Knee Arthroplasty: An Analysis of Neighborhood Deprivation Indices	2024	■ A higher SVI and ADI were associated with increased incidences of ED visits and readmissions postoperatively ■ Patients with higher SVI and ADI were associated with pulmonary embolism and long-term aseptic revision complications
Lulla V Kiwinda	The Effect of Social Drivers of Health on 90-Day Readmission Rates and Costs After Primary Total Hip and Total Knee Arthroplasty	2025	■ Patients with higher levels of ADI had higher likelihood of 90-day unplanned readmission after hospital discharge
Anoop S Chandrashekar	Socioeconomic Indices Are Associated With Increased Resource Utilizations, but Not 90-Day Complications Following Total Hip and Knee Arthroplasty	2025	■ ADI and DCI were not associated with the occurrence of any 90-day complication, reoperation, or revision
Samantha N Baxter	Social Vulnerability adversely affects emergency-department utilization but not patient-reported outcomes after total joint arthroplasty	2024	■ Patients undergoing TKA and THA with higher levels of SVI had higher returns to the ED within 90-days of discharge
Allen C Norris	Social Needs of Patients Undergoing Total Joint Arthroplasty	2022	■ For patients with total joint arthroplasty, ADI scores were positively associated with socials needs at the state and national levels ■ Patients with socials needs had higher 90-day readmissions
Joydeep Baidya	Social determinants of health in patients undergoing hemiarthroplasty: are they associated with medical complications, healthcare utilization, and payments for care?	2023	■ Patients with high ADI had greater medical complications including surgical site infections, cerebrovascular accidents and respiratory failures ■ ADI was not associated with rates of ED visits but patients with higher ADI were readmitted at diminished rates
Matthew J Hadad	Racial Disparities in Outcomes After THA and TKA Are Substantially Mediated by Socioeconomic Disadvantage Both in Black and White Patients	2023	■ ADI did not mediate the associations observed between race and 90-day readmission and ED admission in patients with total knee arthroplasty
Jeremy A Dubin	Race Associated With Increased Complication Rates After Total Knee Arthroplasty	2023	■ ADI was not associated with likelihood of 30-day emergency department visits and readmissions
Anton Khlopas	Neighborhood Socioeconomic Disadvantages Associated With Prolonged Lengths of Stay, Nonhome Discharges, and 90-Day Readmissions After Total Knee Arthroplasty	2022	■ Patients with higher ADI were associated with all-cause readmissions for patients with total knee arthroplasty ■ There were no significant associations between ADI and 90-day emergency department visits or reoperations
Sandeep S Bains	Neighborhood socioeconomic disadvantages associated with increased rates of revisions, readmissions, and complications after total joint arthroplasty	2024	■ Higher ADI was independently associated with increased total complications ■ ADI was not associated with increased total complications at 30 days or 1-year
Benjamin E Jevnikar	Neighborhood Socioeconomic Disadvantage is Associated With Increased Health Care Utilization After Septic and Aseptic Revision Total Hip Arthroplasty	2025	■ Patients with higher ADI had higher ED readmission within 90 days of surgery
Juan D Lizcano	Health Disparities in Aseptic Revision Total Hip Arthroplasty: Assessing the Impact of Social Determinants of Health	2025	■ Patients with higher ADI were revised for aseptic loosening at higher proportion than their counterparts ■ There was lower prevalence of osteolysis in patients with higher ADIs ■ High overall SVI was associated with increased mortality and reoperations ■ Patients with high housing and transportation SVI had higher odds of 90-day complications and prosthetic joint infection episodes
Genna R Potter	How Many Patients Qualify for Extended Oral Antibiotic Prophylaxis Infection Following Primary and Revision Hip and Knee Arthroplasties	2024	■ The mean ADI for high-risk patients was higher than for standard risk patients for patients with total joint arthroplasty being considered for risk of prosthetic joint infections
Victoria S Wu	Impact of social disadvantage among total knee arthroplasty places of service on procedural volume: a nationwide Medicare analysis	2023	■ With each increase in ADI quintile, there was significantly lower total knee arthroplasty utilization
Davis A Hartnett	Socioeconomic Disparities in the Utilization of Total Hip Arthroplasty	2022	■ Patients with increased social deprivation have lower odds of total hip arthroplasty despite hip osteoarthritis diagnoses

Of the 18 studies that directly assessed clinical outcomes, multiple large database studies using ADI reported higher rates and/or odds of medical complications after arthroplasty among patients from high-deprivation areas [2, 5, 10]. While a number of studies found higher 90-day emergency department utilization in patients with higher levels of deprivation [5, 10–13], others reported no association between ADI and ED visits within 90 days specifically following TKA [14, 15]. The association between social deprivation with readmission following arthroplasty was similarly conflicted; some studies reported increased likelihood of 90-day unplanned readmission, while others reported no differences in readmission based on ADI scores [2, 5, 10, 15–17].

### Resource Utilization, Length of Stay, and Discharge Disposition

A total of 13 articles were included which examined the associations between social deprivation and healthcare resource utilization, hospital length of stay, and discharge disposition (Table 3). These studies found that social deprivation was commonly associated with increased spending and payments. Gordon et al. found that higher ADI was associated with increased 90-day expenditures after TKA [5]. Higher payments on the day of surgery and within 90 days were also reported among patients with higher levels of ADI who undergoing hemiarthroplasty [2]. Another study similarly linked higher ADI with higher cost of care after both total hip and total knee arthroplasty [16]. Across multiple articles, higher deprivation was also associated with longer length of hospital stay [6, 7, 11, 15, 18, 19]. However, one study comparing indices reported that length of stay was associated with ADI but not SDI or SVI and another THA-focused analysis found SVI was not associated with 30-day costs or length of stay [6, 20].

**Table 3:** Studies assessing social deprivation and resource utilization, length of stay, and discharge disposition in patients undergoing total joint arthroplasty (*n* = 13)

First author		Year	Key Findings
Adam M Gordon	A Nationwide Analysis of the Impact of Socioeconomic Status on Complications and Health Care Utilizations After Total Knee Arthroplasty Using the Area Deprivation Index: Consideration of the Disadvantaged Patient	2024	■ Patients with higher ADIs had higher 90-day expenditures ($15,066 versus $12,459) than those with lower ADIs
Michelle R Shimizu	Are social determinants of health associated with an increased length of hospitalization after revision total hip and knee arthroplasty? A comparison study of social deprivation indices	2024	■ Patients with higher ADI had more prolonged length of stay following surgery ■ There was no association of length of stay with SDI and SVI
Jeremy Dubin	Assessing social disparities in inpatient vs. outpatient arthroplasty: a in-state database analysis	2024	■ Patients who had overall higher SVI scores had higher levels of inpatient arthroplasty
Michelle R Shimizu	The Utility of Neighborhood Social Vulnerability Indices in Predicting Non-Home Discharge Disposition Following Revision Total Joint Arthroplasty: A Comparison Study	2025	■ Patients in the highest ADI and SDI quartiles had higher odds of non-home discharge ■ Discharge disposition was more strongly associated with ADI than SDI ■ There was no association between SVI and discharge disposition
Tahsin M Rahman	The Impact of Social Determinants of Health on Outcomes and Complications After Total Knee Arthroplasty: An Analysis of Neighborhood Deprivation Indices	2024	■ Patients with higher SVI and ADI had increased rate of discharge to a skilled nursing facility ■ Higher levels of ADI and SVI were associated with greater length of stay
Lulla V Kiwinda	The Effect of Social Drivers of Health on 90-Day Readmission Rates and Costs After Primary Total Hip and Total Knee Arthroplasty	2025	■ Higher levels of ADI were associated with high cost of care
Anoop S Chandrashekar	Socioeconomic Indices Are Associated With Increased Resource Utilizations, but Not 90-Day Complications Following Total Hip and Knee Arthroplasty	2025	■ Patients with higher ADI and DCI had higher levels of increased length of stay
Ronald E Delanois	Social Determinants of Health in Total Hip Arthroplasty: Are They Associated With Costs, Lengths of Stay, and Patient Reported Outcomes?	2022	■ SVI was not associated with 30-day costs and length of stay for patients undergoing total hip arthroplasty
Joydeep Baidya	Social determinants of health in patients undergoing hemiarthroplasty: are they associated with medical complications, healthcare utilization, and payments for care?	2023	■ Patients with higher ADI had significantly higher payments on the day of surgery as well as within 90 days after surgery
Matthew J Hadad	Racial Disparities in Outcomes After THA and TKA Are Substantially Mediated by Socioeconomic Disadvantage Both in Black and White Patients	2023	■ ADI partially mediated the association between race and length of stay of three days or more and the association between race and nonhome discharge
Anton Khlopas	Neighborhood Socioeconomic Disadvantages Associated With Prolonged Lengths of Stay, Nonhome Discharges, and 90-Day Readmissions After Total Knee Arthroplasty	2022	■ Patients with high ADI had higher lengths of stay and non-home discharges for patients who underwent total knee arthroplasty
Benjamin E Jevnikar	Neighborhood Socioeconomic Disadvantage is Associated With Increased Health Care Utilization After Septic and Aseptic Revision Total Hip Arthroplasty	2025	■ Higher ADI was associated with nonhome discharge and extended length of stay
Daniel Grits	Neighborhood Socioeconomic Disadvantage Associated With Increased Healthcare Utilization After Total Hip Arthroplasty	2022	■ Patients with higher ADI had statistically significant higher odds of nonhome discharge and prolonged length of stay

Discharge disposition demonstrated one of the most consistent relationships with deprivation. Patients with higher levels of deprivation had higher likelihood of non-home discharge following both primary and revision arthroplasty [11, 15, 18, 19, 21]. In a comparative analysis of deprivation indices within a revision arthroplasty cohort, discharge disposition was more strongly associated with ADI than SDI and not associated with SVI [21]. In another study, higher SVI was associated with greater likelihood of inpatient arthroplasty in an in-state database analysis [4].

### Patient-Reported Outcomes and Functional Recovery

Seven studies evaluated deprivation indices in relation to patient-reported outcomes and functional recovery after arthroplasty (Table 4). Results from these studies were mixed. Some studies showed no association between deprivation and 1-year change in pain or function after TKA, and ADI was not associated with long-term opioid use following TKA [22, 23]. Likewise, in THA, ADI was not associated with failure to achieve the minimal clinically important difference (MCID) as measured by the hip disability and osteoarthritis outcome score (HOOS) pain in one study, and in TKA, ADI was not associated with inability to achieve MCID for selected knee injury and osteoarthritis outcome score (KOOS) domains [24, 25].

**Table 4:** Studies assessing social deprivation and patient-reported outcomes and functional recovery in patients undergoing total joint arthroplasty (*n* = 7)

First author		Year	Key Findings
Sarah H Yi	Community-Level Social Vulnerability and Hip and Knee Joint Replacement Surgery Receipt Among Medicare Enrollees With Arthritis	2024	■ Patients with higher SVI had lower total joint arthroplasty overall
Nicholas R Pagani	Socioeconomic Disadvantage Predicts Decreased Likelihood of Maintaining a Functional Knee Arthroplasty Following Treatment for Prosthetic Joint Infection	2024	■ The proportion of patients who had a functional knee arthroplasty declined significantly with increasing ADI for patients who had TKA for prosthetic joint infection
Elizabeth C Danielson	Social risk and patient-reported outcomes after total knee replacement: Implications for Medicare policy	2024	■ There were no associations between county-level social vulnerability and change in pain or function for one year-outcomes after total knee arthroplasty
Katherine Hadlandsmyth	Social Determinants of Long-Term Opioid Use Following Total Knee Arthroplasty	2024	■ ADI was not associated with long-term opioid use for patients undergoing total knee arthroplasty
Brian Benyamini	Neighborhood Socioeconomic Disadvantage May Influence 1-Year Patient-Reported Outcome Measures After Total Hip Arthroplasty	2025	■ ADI was not associated with failure to achieve minimal clinical important difference in the Hip Disability and Osteoarthritis Outcome Score Pain ■ Patients with higher ADI scores had increased odds of failing to achive patient acceptable symptom state for Hip Disability and Osteoarthritis Outcome Score Pain, Physical Function Shortform (PS) and Joint Replacement (JR) ■ ADI was not associated with failure to achieve patient satisfaction at 1 year
Matthew J Hadad	High Area Deprivation Index is Associated With Not Achieving the Patient-acceptable Symptom State After TKA	2024	■ ADI was not associated with inability to achieve the minimum clinically important difference for the Knee Injury and Osteoarthritis Outcome Score for pain, for Joint Replacement, but not for Physical Function
Pedro J Rullan	How to Raise the Bar in the Capture of Patient-Reported Outcome Measures in Total Joint Arthroplasty	2024	■ Residence with a higher ADI was associated with requiring active follow-up after total joint arthroplasty

In contrast, other analyses suggest that higher deprivation is associated with decreased achievement of “acceptable” recovery thresholds. After THA, higher ADI was associated with higher likelihood of failing to achieve patient acceptable symptom state (PASS) across multiple HOOS domains. After TKA, ADI was associated with not achieving PASS for KOOS physical function [24, 25]. In the setting of prosthetic joint infection, increasing ADI was associated with a declining proportion of patients maintaining a functional knee arthroplasty [26]. Finally, one study highlighted that residence in higher ADI areas was associated with requiring active follow-up to capture patient-reported outcomes, suggesting differential follow-up burden in more deprived populations [27].

## Discussion

### Access to Arthroplasty and 30–90 Day Clinical Outcomes

The results of this study suggest that social deprivation, as measured by ADI, SVI, SDI and DCI, is associated with decreased access to arthroplasty and worse early postoperative outcomes [5, 10, 11, 28, 29]. Several studies also demonstrated greater healthcare utilization among patients from more deprived communities, reflecting increased need for postoperative resource support [5, 7, 12, 18]. Together, these findings underscore the important role of social determinants of health in shaping early recovery after arthroplasty.

This study also found that high levels of deprivation were consistently linked to higher rates of postoperative medical complications. Patients residing in areas of high deprivation indices experienced acute kidney injury, respiratory failure, infection, and other medical complications at greater incidences within a 90-day postoperative period [2, 5, 10, 29]. Even after adjusting for comorbidities and perioperative risks, associations with increased postoperative complications persisted. This indicates that deprivation is a predictor for adverse postoperative experiences for patients independent of extraneous patient risk factors [28, 29]. Notably, patients within higher-deprivation cohorts were more likely to be female, Black, and from lower-income households, highlighting the intersection of socioeconomic disadvantage with broader structural inequities that may influence surgical outcomes [4, 25].

Another consistent theme throughout the literature is the resource strain and burden that patients with higher deprivation place on healthcare systems following TJA [7, 17, 18]. Patients from higher deprivation areas were seen to have higher rates of emergency department (ED) visits within 90 days after the index procedure [2, 4, 5, 10]. However, while the incidence of an ED visit after surgery reliably increased within the higher deprivation groups, there was not a reliable increase in hospital readmission. Readmission rates remained unchanged or lower among higher deprivation groups. These incongruous findings raise the question of other contributing factors such as limited access to adequate inpatient care or lower reliability of ED service within higher deprivation areas [7, 14].

These findings highlight the importance of integrating social deprivation evaluations for preoperative risk mitigation and perioperative planning. Social screening, targeted interventions, medication cost support, and discharge care should be offered as a strategy to combat disparities among deprivation groups seeking TJA. In this way, physicians and surgeons can address needs of patients prior to surgical interventions and before complications arise.

### Resource Utilization, Length of Stay, and Discharge Disposition

The comprehensive data in these studies, measured by ADI, SVI, SDI, and DCI, yield mixed results regarding the relationships among resource utilization, length of stay, and discharge disposition. However, social deprivation measured by ADI is a significant factor impacting healthcare expenditures, hospital length of stay, and discharge disposition [2, 6, 7, 10, 16]. These findings highlight the importance of incorporating social determinants into postoperative care protocols to ensure equity across patient populations.

The aggregate data from these studies suggest that ADI is the most consistent in assessing associations between resource utilization and social deprivation in arthroplasty patients [2, 15, 18, 19]. Patients with high ADI scores consistently demonstrated higher postoperative costs, prolonged hospital stays, and increased odds of non-home discharge. In contrast, other deprivation measures, such as the SDI and SVI, had more inconsistent associations with healthcare utilization, length of stay, and discharge disposition [4, 20, 21].

Socially deprived patients had longer length of hospital stays as well as non-home discharges to skilled nursing facilities and rehabilitaiton centers [15]. These observed findings may be attributed to restricted or unstable housing which preclude recovery following TJA. Furthermore, increased length of stay and non-home discharge have a disproportionally negative economic impact on patients from higher deprivation areas, with higher deprivation patients reporting a higher 90-day recovery expenditure [7, 15–17]. Hence, patients within that higher deprivation cohort reported a higher 90-day recovery expenditure. It is in the best interest of the healthcare system to better understand the needs of socially deprived populations in order to reduce healthcare costs.

These associations suggest the need for enhanced preoperative risk stratification and tailored postoperative discharge planning to ensure that patients with high ADI receive adequate care and mitigate cost disparities. However, it is imperative to note that this data explicitly evaluates the association between social deprivation and higher length of stay, non-home discharge, and costs after surgery, rather than the disparities in clinical outcomes.

### Patient-Reported Outcomes and Functional Recovery

Compared with utilization, discharge disposition, and early healthcare use, associations between social deprivation and patient-reported outcomes after total joint arthroplasty are less consistent. Several studies demonstrated no association between deprivation indices and achievement of MCID in pain or function following total hip or knee arthroplasty, suggesting that socially deprived patients can experience meaningful symptomatic improvement after surgery [22, 24, 25]. Similarly, social deprivation was not associated with long-term opioid use following total knee arthroplasty [23].

In contrast, when outcomes were evaluated using absolute recovery thresholds, higher deprivation was more consistently associated with worse outcomes. Multiple studies report that higher ADI scores are associated with increased odds of failing to achieve PASS for pain and functional domains after arthroplasty [24, 25]. This divergence between MCID and PASS suggests that although deprived patients may improve relative to baseline, they may be less likely to reach symptom levels perceived as satisfactory, potentially due to lower preoperative function or greater postoperative barriers.

The impact of deprivation appears more pronounced in higher-risk or complex populations. Among patients treated for prosthetic joint infection, increasing ADI was associated with a declining likelihood of maintaining a functional knee arthroplasty [26]. Additionally, patients from more deprived neighborhoods were more likely to require active follow-up to capture outcomes, indicating potential challenges with longitudinal outcome assessment and possible underrepresentation [27].

Overall, these findings suggest that social deprivation does not preclude clinically meaningful improvement after arthroplasty but may limit the likelihood of achieving optimal functional recovery. Future research should distinguish between relative improvement and absolute recovery thresholds and account for disparities when evaluating patient-reported outcomes across socioeconomic contexts.

## Conclusion

Social deprivation is an important factor influencing access, early outcomes, and healthcare utilization after total joint arthroplasty. Among commonly used composite measures, the Area Deprivation Index (ADI) demonstrated the most consistent and robust associations across multiple domains, including lower arthroplasty utilization, increased postoperative complications, longer hospital stays, higher costs, and greater likelihood of non-home discharge. While socially deprived patients generally achieved meaningful improvements in pain and function, they were less likely to reach patient-acceptable recovery thresholds.

Incorporating social deprivation measures into preoperative risk assessment and postoperative planning may help identify patients who would benefit from targeted support. Greater standardization in the use of deprivation indices, along with prospective evaluation of tailored interventions, is needed to reduce disparities and optimize arthroplasty outcomes.

## Key References

- Gordon A, Vakharia R, Mont M. A nationwide analysis of the impact of socioeconomic status on outcomes after total knee arthroplasty using the Area Deprivation Index: consideration of the disadvantaged patient. Aging Clin Exp Res. 2024;36:S135–6. 
  - Large, contemporary, national analysis linking neighborhood deprivation (ADI) to multiple early postoperative outcomes after TKA, including medical complications, emergency department utilization, readmissions, and costs.
- Khlopas A, Grits D, Sax OC, Chen Z, Orr MN, Klika AK, et al. Neighborhood Socioeconomic Disadvantages Associated With Prolonged Lengths of Stay, Nonhome Discharges, and 90-Day Readmissions After Total Knee Arthroplasty. J Arthroplasty. 2022;37(6):S37. 
  - Arthroplasty-focused study that clearly demonstrates a graded association between higher ADI and clinically meaningful utilization endpoints (prolonged length of stay, non-home discharge, and 90-day readmissions) after TKA.
- Rollings KA, Noppert GA, Griggs JJ, Ibrahim AM, Clarke PJ. Comparing Deprivation vs Vulnerability Index Performance Using Medicare Beneficiary Surgical Outcomes. JAMA Surg. 2024;159(12):1404–13. 
  - This paper helps contextualize why different deprivation measures may yield different associations and supports more standardized, intentional index selection in future arthroplasty disparities research.

## Data Availability

No datasets were generated or analysed during the current study.
